# Organizational Climate Factors Influencing Job Satisfaction in Rural Health Care Workplaces in Upper-Middle-Income and High-Income Countries: A Scoping Review

**DOI:** 10.3390/healthcare14091238

**Published:** 2026-05-04

**Authors:** Ginger E. Minahan, Sandra C. Thompson

**Affiliations:** 1 Department of Global Health, Georgetown University, Washington, DC 20057, USA; 2 Western Australian Centre for Rural Health, University of Western Australia, Geraldton, WA 6530, Australia; sandra.thompson@uwa.edu.au

**Keywords:** job satisfaction, organizational climate, retention, rural

## Abstract

Background: Fostering job satisfaction in rural health workplaces is crucial due to its relationship with multiple outcomes such as physical and mental health, job performance, burnout, turnover intention, and retention. As knowledge of the organizational climate components which influence rural job satisfaction is currently limited, this scoping review sought to identify organizational climate factors influencing job satisfaction among the rural health care workforce in upper-middle-income and high-income countries, organized by thematic categories for use in future workplace initiatives. Methods: Following JBI scoping review methodology and the PRISMA-ScR checklist, studies published between January 2013 and October 2023 were identified through searches of PubMed, APA PsycINFO, CINAHL, and Google Scholar. Eligible studies reported research evaluating organizational climate factors as facilitators of and/or barriers to job satisfaction among health care workers in rural regions of upper-middle-income and high-income countries. Screening was conducted in Covidence and data were extracted using a customized Excel tool. Results: Of 305 identified articles that underwent screening, eighteen studies were included in the final review. Thirteen components of organizational climate across studies were identified as facilitators of and/or barriers to job satisfaction in rural health care workplaces, and categorized into four overarching domains: interpersonal relationships, individual responsibilities, organizational structure and planning, and reward and professional development. Conclusions: Relationships with leadership, peer relationships, autonomy and freedom of working method, availability and quality of resources, workload and the ability to remain busy, and wages and benefits emerged as the most consistently identified components impacting job satisfaction across studies. Rural health care organizations are encouraged to prioritize these components and implement participatory, communicative leadership structures, foster team cohesion and support, develop health care worker autonomy and limit inefficient oversight structures, address resource gaps, coordinate with workers to determine manageable workloads, and implement competitive wages and benefits. Additional research is needed to better elucidate the associations between job satisfaction and organizational climate components as well as to longitudinally evaluate interventions aiming to foster rural health workers’ job satisfaction.

## 1. Introduction

Job satisfaction is a “pleasurable or positive emotional state resulting from the appraisal of one’s job or job experiences”, and has proven influential in multiple studies establishing relation between health care workers’ job satisfaction and physical and mental health, patient satisfaction, job performance, burnout, turnover intention, as well as retention [1,2,3,4,5,6,7,8].

Despite a global trend towards urbanization, nearly half (43%) of the world’s population remain in rural areas [9]. In addition to frequent barriers to health care such as physical distance from health services, socioeconomic challenges, or stigma of health conditions, rural populations experience disproportionately poorer health outcomes compared to urban populations, in part due to difficulties retaining health care workers in rural regions [10,11,12]. As poor retention of rural health care workers is a threat globally, understanding factors that influence job satisfaction and hence retention is critical [1,13,14].

Organizational climate is a “general concept constituted by employees’ subjective opinions about their organization, management, and other environmental factors. It represents a group of attributes that are used to describe an organization’s behaviour” [15]. As organizations can exert influence over several aspects of an individual’s work, organizational climate can play a substantial role in job satisfaction. Previous reviews have identified organizational factors of job satisfaction, although in exclusively non-rural and profession-specific contexts. Specchia et al. [16] reported a significant positive correlation between a transformational leadership style and nurses’ job satisfaction, Penconek et al. [17] found significant positive relationships between autonomy, social support among team members, and job satisfaction of nurse managers, and Hayes [18] reported that nurses’ job satisfaction was influenced by factors such as autonomy, colleague interaction, organizational policies, resource availability, and educational opportunities. In contrast, Aloisio et al. [19] found no significant associations between long-term care nurses’ job satisfaction and organizational factors including facility ownership, supervisor and manager support, resources, staffing level, and social relationships, a finding likely reflective of the distinctive nature of long-term care settings. No review has yet examined organizational climate factors of job satisfaction in rural settings among multiple professional groups, excluding a substantial proportion of the health workforce. Examining rural workforce experiences has particular importance given longstanding problems related to recruitment and retention in rural settings, with workforce turnover significantly impacted by job satisfaction [12]. Focusing on rural health care workers in research is crucial to identify key areas for intervention. Previous reviews have additionally focused on nurses’ job satisfaction, however ongoing retention issues occur for a range of health care workers across rural settings including nurses, physicians, advanced practice providers, and allied health staff [12].

This scoping review was undertaken to identify and summarize international literature concerning organizational climate factors which influence job satisfaction in rural health care workplaces in upper-middle-income and high-income countries, organizing identified factors into thematic categories to assist planning and implementation of future workplace initiatives. Taking into consideration the lack of comprehensive occupational safety and health (OSH) measures in many lower-middle-income and low-income countries, as well as the impact OSH measures have on perceived occupational health risks and ultimately organizational climate and job satisfaction, this review focuses on upper-middle-income and high-income countries to improve the comparability of the studied workplace environments [20,21].

## 2. Review Question

The review question was developed using the PCC (population/concept/context) framework model by Joanna Briggs Institute (JBI) [22]. We broadly identified the population as the health care workforce, concept as organizational climate components of job satisfaction, and context as rural regions within upper-middle-income and high-income countries. To meet the objective of the review, we asked the question: What components of organizational climate influence job satisfaction in rural health care workplaces in upper-middle-income and high-income countries?

## 3. Methods

### 3.1. Study Design

This scoping review was conducted in accordance with JBI methodology for scoping reviews and guided by the Preferred Reporting Items for Systematic reviews and Meta-Analyses extension for Scoping review (PRISMA-ScR) checklist (See Supplementary Material S1) [22,23].

### 3.2. Eligibility Criteria

Eligibility criteria were developed using the PCC framework. Studies were included and excluded based on the following criteria in Box 1:

Box 1 **Eligibility criteria.**

**Inclusion****Exclusion**Population-Health care workers -Participants aged ≥18 years-Non-health care workers -Health students/registrars -Participants aged <18 yearsConcept-Organizational climate facilitators of and barriers to job satisfaction Organizational climate defined as a “general concept constituted by employees’ subjective opinions about their organization, management, and other environmental factors” [15]Job satisfaction defined as: “pleasurable or positive emotional state resulting from the appraisal of one’s job or job experiences” [5]-Non-organizational climate facilitators of and barriers to job satisfaction -Health care workers’ satisfaction in a preceptor/supervisor role -Online “remote” work/telemedicineContext-Rural areasDefined as areas of low population size, population density, or geographic distance from an urban region [24]-Upper-middle-income and high-income countriesDefined using World Bank country classifications [25]-Urban regions -Lower-middle-income and low-income countriesTypes of sources-Primary research/secondary analysis -Studies published within last 10 years (1 January 2013—search date) -Studies reported in English language -Studies about Human species-Literature reviews/meta-analyses/textbooks/dissertations/editorials -Studies published before 2013 -Non-English language studies -Studies about non-human species

### 3.3. Search Strategy

With the aid of a health librarian, an initial restricted search of PubMed was undertaken to identify relevant sources. Key words contained in the titles and abstracts of relevant articles were used to develop a full search strategy (See Supplementary Material S2). The search strategy involved four primary concepts: “health personnel”, “job satisfaction”, “rural”, and “organizational climate”. We combined these search terms using the Boolean operator “AND”. Four bibliographic databases were searched: PubMed, APA PsycINFO, CINAHL, and Google Scholar. Search terms were adjusted according to the search methods of each database. In response to rapid changes in contemporary health care workplaces—such as use of new technologies, epidemiological shifts, and consecutive work hour policies—searches only included articles published in the past ten years to maximize their relevance [26,27]. Filtering methods were consistent across databases except Google Scholar, including date range (publication within the last ten years), English language, and Human species specification. Google Scholar filtering methods allowed only for date range specification (publication within the last ten years). The search of Google Scholar was limited to the first ten pages, as upon preliminary inspection, results beyond page ten were consistently unrelated to the review question and deemed irrelevant. All final searches were conducted on 24 October 2023.

### 3.4. Source of Evidence Selection

Following the searches, all retrieved sources were uploaded into Zotero. The sources were then transferred to Covidence, where duplicate articles were removed and two or more authors screened the titles and abstracts of articles for relevance. Eligibility criteria were used to assess the relevance of the articles. Any disagreements that arose between authors at this stage of screening were resolved through discussion. Relevant articles identified in the title and abstract screening stage were screened for full-text review by one independent author (GM), with regular discussion supplemented by solicited review where the primary author was hesitant about a paper’s relevance. Eligibility criteria were used to assess the relevance of these articles, with studies excluded if there was insufficient analysis of rurality, job satisfaction, and organizational climate. A PRISMA flow diagram details the results of the search and reasons for exclusion [28].

### 3.5. Data Extraction

A data extraction tool was collaboratively customized in Excel by the authors to meet the objective of this review. The data from sources meeting inclusion criteria were extracted by one author (GM) and separated into qualitative and quantitative key findings tables. The second reviewer (ST) thoroughly checked all data extracted for the first five papers undergoing extraction with strong agreement between authors, and regular discussion and review additionally occurred throughout the data extraction stage to ensure rigor and consistency.

### 3.6. Quality Assessment

No quality assessment was completed in accordance with JBI scoping review methodology, which does not mandate formal appraisal for scoping reviews [22].

### 3.7. Data Analysis

Data analysis was conducted by one independent author using a thematic analysis approach, with findings validated through iterative discussion with the second author. After compiling organizational climate contributors to job satisfaction across studies, the author identified recurrent reported organizational climate components by conducting a frequency analysis to determine how many studies included each particular component. Components were considered “recurrent” if identified in three or more studies. These recurrent components were treated as deductive codes, evaluated for meaningful themes, and further refined into four key domains capable of encompassing all identified organizational climate facilitators of and/or barriers to job satisfaction. As components meeting the minimum threshold of three or more studies vary in their evidentiary support, emphasis is placed on prioritizing components identified across eight or more studies. The authors have reviewed and edited the output and take full responsibility for its accuracy. With this exception, no AI tool was used at any stage of data collection, screening, extraction, or analysis.

## 4. Results

**Figure 1:**
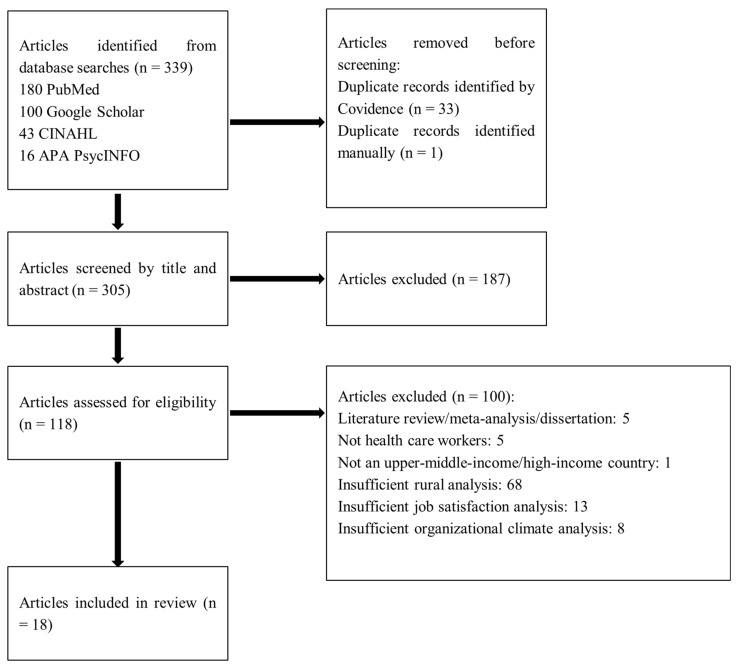
PRISMA flow diagram.

The PRISMA flow diagram (Figure 1) details the search and selection of relevant studies [28]. Of 339 identified articles, after removal of duplicates, 305 were screened at the title and abstract stage, and 118 met criteria for full-text review. Ultimately, 18 articles from the full-text review stage were eligible and included in the review.

### 4.1. Characteristics of Studies

The characteristics of included articles are outlined in Table 1 and Table 2. The majority of the studies were conducted in Australia (*n* = 5, 28%), China (*n* = 4, 22%), and the United States (*n* = 4, 22%). The remaining studies were conducted in Brazil (*n* = 1, 6%), Serbia (*n* = 1, 6%), Ecuador (*n* = 1, 6%), Germany (*n* = 1, 6%), and Japan (*n* = 1, 6%). Six studies (33%) were qualitative and utilized either semi-structured interviews (*n* = 5) or open-ended interviews (*n* = 1) to collect data. Twelve studies (67%) were quantitative and utilized self-report surveys (*n* = 12) to collect data. Populations included in these studies can broadly be divided into physicians (*n* = 11, 61%), nurses (*n* = 9, 50%), allied health staff (*n* = 4, 22%), advanced practice providers (physician assistants and nurse practitioners) (*n* = 3, 17%), and administrative health staff (*n* = 1, 6%). As language used to describe the roles of health professionals varies internationally, population descriptions as written in the articles can be found in Table 1 and Table 2.

### 4.2. Organizational Climate Components of Job Satisfaction

**Figure 2:**
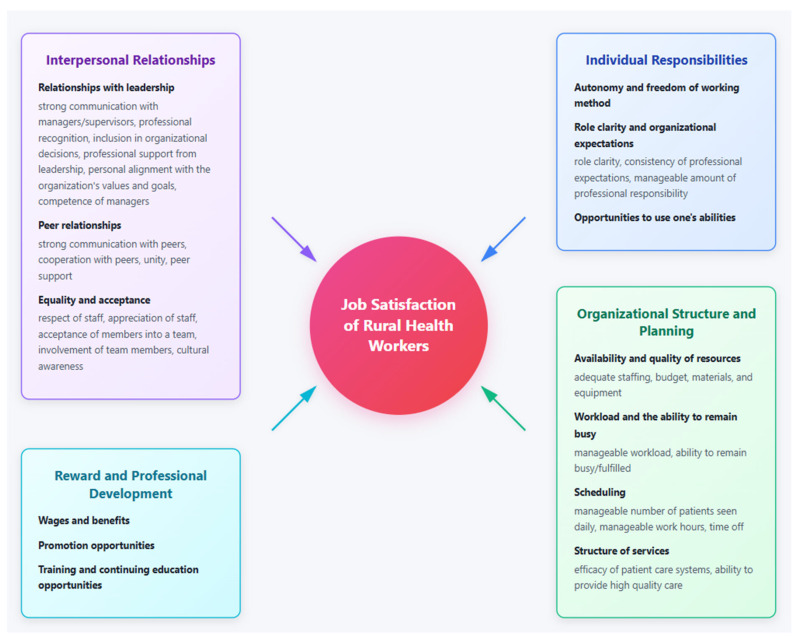
Key organizational climate components of job satisfaction.

Four overarching categories of organizational climate were identified as contributors to job satisfaction in rural health care workplaces: interpersonal relationships (*n* = 18 papers), individual responsibilities (*n* = 11), organizational structure and planning (*n* = 15), and reward and professional development (*n* = 12). Thirteen components of organizational climate were established within these categories based on recurring themes reported in both qualitative and quantitative research: relationships with leadership, peer relationships, equality and acceptance, autonomy and freedom of working method, role clarity and organizational expectations, opportunities to use one’s abilities, the availability and quality of resources, workload and the ability to remain busy, scheduling, the structure of services, wages and benefits, promotion opportunities, and training and continuing education opportunities. Components and sub-components were found to function as facilitators of and/or barriers to job satisfaction depending on their presence or scale, and their influence specific to each study is shown in Table 1 and Table 2. Figure 2 shows a visual summary of the identified categories and components.

#### 4.2.1. Interpersonal Relationships

Relationships with leadership was the most frequently mentioned component of organizational climate influencing job satisfaction in rural health care workplaces (*n* = 16), with sub-components found to facilitate or hinder job satisfaction including communication practices with managers and supervisors, professional recognition, inclusion in organizational decisions, competency of leadership in decision-making, professional support from leadership, and the values and goals of organizations [29,30,31,32,33,34,35,36,37,38,39,40,41,42,43,44]. For example, Bragg and Bonner [29] described the influence of “value alignment” among Australian nurses and their organization: shared values regarding the organizational support and resources required to produce a high standard of care, including sufficient communication with leadership, contributed to job satisfaction. A divergence of values between nurses and leadership, manifesting in differing priorities such as nurses’ value of high standard of care compared to organizations’ focus on low expenditure, contributed to dissatisfaction due to a decline in quality of care. Satisfaction with leadership was rated highly across multiple studies: doctors in China surveyed in Fang et al. [32], using a 5-point scale, were most satisfied with the competence of their managers in decision-making (Mean ± SD = 2.87 ± 0.82) and the manner in which their boss managed workers (Mean ± SD = 2.76 ± 0.86). Physician assistants (PAs) in the United States surveyed in Filipova [33] were most satisfied with authority, reporting a mean satisfaction level of 4.48 ± 0.55 using a 5-point scale. A significant positive association between job support from managers and job satisfaction for Australian nurse consultants (NCs) was further established in Giles et al. [34] (β = 0.27, t = 3.02, *p* < 0.01, CI = 0.05, 0.24).

Fourteen studies explored the influence of peer relationships on job satisfaction, and described sub-components of peer relationships including communication with peers, cooperation with peers, unity, and peer support as facilitators of or barriers to job satisfaction [30,31,33,35,36,37,38,39,40,42,43,44,45,46]. De Oliveira et al. [31] found teamwork, involving peer cooperation, support, and unity to facilitate job satisfaction among nurses in Brazil, while a lack of peer support in addition to isolation from other staff members within the office contributed to decreased job satisfaction for Australian Aboriginal mental health workers (AMHWs) studied in Cosgrave et al. [30]. Hartung and Miller [37] identified a sense of emotional closeness among nurses in the United States as a contributor to job satisfaction, however also found that the geographic distribution of rural workers induced occasional isolation or loneliness which can impact the efficiency of communication practices and overall job satisfaction. Health care workers frequently rated satisfaction with peer relationships highly: 91.1% of Australian junior doctors surveyed in Lennon et al. [39] reported satisfaction with their colleagues. Leutgeb et al. [40], using a 7-point scale, found GPs in Germany to be most satisfied with colleagues (Mean ± SD = 5.28 ± 1.2), and Izquierdo-Condoy [38] reported physicians in Ecuador to experience their highest satisfaction relating to participation in departmental decisions (Mean ± SD = 4.5 ± 1.6). Primary care providers (PCPs) in China, surveyed in Wang et al. [44] using a 5-point scale, experienced high job satisfaction with the medical practicing environment, including peer relationships and support (Mean ± SD = 3.81 ± 0.64). However, low satisfaction with a cooperative working environment was identified in Grujicic et al. [35], with physicians and nurses in Serbia reporting a mean satisfaction score of 2.99 ± 1.24. Gu et al. [36] established a significant association between job satisfaction and peer relationships for physicians in China (β = 0.36, *p* < 0.001), and Waddimba et al. [46] reported an association between gratified relatedness needs, including peer support, and increased frequency of job satisfaction for practitioners in the United States (b = 0.19, t = 2.49, *p* = 0.01, CI = 0.04, 0.34).

Five studies evaluated the role of equality and acceptance in job satisfaction, and specifically identified sub-components of respect, appreciation of staff, acceptance of members into a team, involvement of team members, and cultural awareness as facilitators of, or barriers to, job satisfaction [29,30,37,38,42]. Cosgrave et al. [30] found that AMHWs in NSW Australia experienced low job satisfaction due to a lack of acceptance and cultural awareness from their team and organization, often receiving limited opportunities for involvement and expectations from leadership to act as “cultural consultants” for Aboriginal clients rather than using the mental health skills they had acquired in training. Physicians in Ecuador surveyed in Izquierdo-Condoy et al. [38] additionally experienced low job satisfaction relating to equality and fairness of treatment based on use of a 7-point scale (Mean ± SD = 3.4 ± 1.8). Australian nurses interviewed in Bragg and Bonner [29] reported job satisfaction when they felt appreciated and respected, and correspondingly experienced increased dissatisfaction as patient care was compromised due partly to attitudes from leadership that nurses were replaceable. Shea [42] found nurse practitioners (NPs) in the United States to also experience a struggle for acceptance in which NPs believed their professional abilities to be challenged and undervalued by physicians, as well as their voices within the organization to be limited due to an implicit provider hierarchy. In an effort to maintain job satisfaction, NPs attempted to counteract the negative actions of leadership in part by continuing to attend staff meetings despite lacking the power to vote. Hartung and Miller [37] identified management and communication techniques undertaken by American nurse managers, such as respect of staffs’ opinions as well as their acceptance of staff to positively contribute to team functioning and overall job satisfaction.

#### 4.2.2. Individual Responsibilities

Eight studies explored the influence of autonomy and freedom of working method on job satisfaction [33,34,35,39,40,42,44,46]. American NPs described as struggling for acceptance in Shea [42] experienced difficulties with consistent physician oversight due to their perceived lack of qualifications, indicating a lack of autonomy which acted as a barrier to NPs’ job satisfaction. Health care workers across multiple studies indicated that they were most satisfied with autonomy and freedom of working method in their respective workplaces: using a 5-point scale, Filipova [33] found PAs in the United States to be most satisfied with autonomy (Mean ± SD = 4.48 ± 0.55) and identified a statistically significant correlation between autonomy and job satisfaction factors. Grujicic et al. [35] found physicians and nurses in Serbia to experience their highest levels of job satisfaction with autonomy (Mean ± SD = 3.61 ± 1.32). Using a 7-point scale, Leutgeb et al. [40] reported that general practitioners (GPs) in Germany rated their satisfaction with freedom of working method highly among the other organizational climate factors studied (Mean ± SD = 5.05 ± 1.5), and a strong correlation was found between job satisfaction and freedom of working method. Nearly two-thirds (65.9%) of Australian junior doctors surveyed in Lennon et al. [39] reported satisfaction with the ability to choose their own work method. Rather than reporting job satisfaction frequency or levels as in the majority of quantitative studies, Waddimba et al. [46] identified that fulfilled autonomy needs were associated with increased variation in job dissatisfaction frequency among practitioners in the United States (b = 0.36, t = 3.76, *p* < 0.001, CI = 0.17, 0.55), and Giles et al. [34] found job autonomy to have a significant positive impact on NCs’ job satisfaction in Australia (β = 0.22, t = 2.51, *p* = 0.01, CI = 0.04, 0.35).

Five studies discussed role clarity and organizational expectations, identifying the impact of role clarity, consistency of professional expectations, and the amount of professional responsibility as facilitators of or barriers to job satisfaction [30,34,39,40,43]. While Cosgrave et al. [30] found that the managers and other staff working with AMHWs in Australia had little understanding of their tasks and responsibilities and this contributed to the AMHWs feeling underworked and less satisfied overall, Tham et al. [43] reported a link between clear role definition and an increase in job satisfaction for primary health care (PHC) staff in Australia. Giles et al. [34] additionally identified role clarity to have a significant positive impact on job satisfaction (β = 0.20, t = 2.19, *p* = 0.03, CI = 0.03, 0.56) for NCs in Australia, whereas role conflict or inconsistent organizational expectations had a significant negative impact on job satisfaction (β = −0.23, t = −2.48, *p* = 0.01, CI = −0.41, −0.05). Lennon et al. [39] determined that 83.2% of Australian junior doctors surveyed were satisfied with their amount of responsibility; Leutgeb et al. [40] similarly found a high level of satisfaction with amount of responsibility among GPs in Germany using a 7-point scale (Mean ± SD = 4.85 ± 1.5), as well as a strong correlation between job satisfaction and amount of responsibility.

Three studies assessed the influence of opportunities to use one’s abilities on job satisfaction [32,39,40]. All three studies found opportunities to use one’s abilities professionally was a facilitator of job satisfaction: Chinese doctors surveyed in Fang et al. [32], using a 5-point scale, rated their satisfaction with the opportunity to make use of their abilities highly among other organizational climate factors (Mean ± SD = 2.81 ± 0.77). Lennon et al. [39] found the majority of junior doctors in Australia (80.0%) were satisfied with their opportunities to use their abilities. Leutgeb et al. [40] described GPs in Germany as likewise moderately satisfied with their opportunity to use their abilities using a 7-point scale (Mean ± SD = 4.89 ± 1.5), and additionally reported a strong correlation between the opportunity to use abilities and job satisfaction.

#### 4.2.3. Organizational Structure and Planning

Eight studies evaluated the influence of availability and quality of resources in job satisfaction, focusing on staffing, budget, and materials and equipment [29,31,33,35,36,37,44,45]. Resources were examined as both a facilitator of and barrier to job satisfaction; Bragg and Bonner [29] found resources such as a high budget or sufficient number of experienced staff to result in increased job satisfaction for nurses in Australia due to a desire for quality patient care; nurses experienced a decrease in job satisfaction when organizations prioritized concerns such as low expenditure. Brazilian nurses surveyed in de Oliveira et al. [31] reported poor physical infrastructure as well as a lack of equipment, medicines, water, and technologies to create challenges in care provision which contributed to decreased job satisfaction. Several studies reported moderate satisfaction with resources in the workplace: using a 5-point scale in Grujicic et al. [35], physicians and nurses in Serbia identified their mean fulfillment or satisfaction with current equipment to be 3.18 ± 1.35, and Wang et al. [44] similarly found PCPs in China to be moderately satisfied with working environment satisfaction, which encompassed work environment, drug need, and equipment need (Mean ± SD = 3.53 ± 0.75). Gu et al. [36] also determined that working conditions, including both medical equipment and the quality of a team, were significantly associated with the job satisfaction of physicians in China (β = 0.34, *p* < 0.001).

Eight studies explored the role of workload and the ability to remain busy in job satisfaction [30,31,32,33,36,40,44,46]. Workload was primarily examined as a barrier to job satisfaction whereas the ability to remain busy was found to be a facilitator. De Oliveira et al. [31] found nurses in Brazil to struggle with decreased job satisfaction from work overload, largely due to additional administrative and bureaucratic responsibilities, whereas Cosgrave et al. [30] found insufficient workload decreased job satisfaction for Australian AMHWs. Chinese doctors surveyed in Fang et al. [32] using a 5-point scale were least satisfied with their workload (Mean ± SD = 1.99 ± 1.09), however were satisfied with their ability to remain busy and fulfilled (Mean ± SD = 2.73 ± 0.82). American PAs surveyed in Filipova [33] using a 5-point scale (Mean ± SD = 3.64 ± 0.77) experienced moderate satisfaction, however were similarly least satisfied with their workload in comparison to other studied organizational climate factors. Gu et al. [36] determined “job description”, including workload, to have a significant association with job satisfaction for physicians in China (β = 0.11, *p* < 0.001). This finding was echoed in Waddimba et al. [46], which found a heavier workload to be associated with a decreased likelihood of frequent job satisfaction (b = −0.34, t = −5.65, *p* < 0.0001, CI = −0.46, −0.22) and frequent job dissatisfaction among practitioners in the United States (b = 0.27, t = 4.55, *p* < 0.0001, CI = 0.15, 0.39).

Five studies examined the impact of scheduling on job satisfaction, detailing patients seen daily, work hours, and time off [35,39,40,42,45]. Poor scheduling acted as a barrier to job satisfaction across studies: Shea [42] reported NPs in the United States were scheduled for a large number of patients daily, leading to decreased job satisfaction due to an inability to deliver the holistic and thorough care desired. NPs worked during lunch and after hours to compensate for their organizations’ limited time slots for patients or to care for non-paying patients in an effort to provide sufficient care and maintain job satisfaction. Leutgeb et al. [40] focused on scheduling specifically in the context of German GPs working in out-of-hours care, or time periods in which regular medical ambulatory services are unavailable. This study found GPs to be least satisfied with their hours of work using a 7-point scale (Mean ± SD = 3.60 ± 1.6) and found a strong correlation between hours of work and job satisfaction. Japanese physicians surveyed in Nojima et al. [45] were moderately satisfied with work hours/conditions using a 4-point scale (Mean ± SD = 1.7 ± 0.9). Lennon et al. [39] determined only 37.9% of junior doctors were satisfied with their work hours and 22.9% were satisfied with their time off, and Grujicic et al. [35], using a 5-point scale, reported physicians and nurses in Serbia to experience low job satisfaction due to inadequate time off (Mean ± SD = 2.07 ± 1.30).

Three studies discussed the structure of services, specifically the restructuring of services and efficacy of patient care systems as facilitators of or barriers to job satisfaction [29,42,43]. Bragg and Bonner [29] identified the restructuring and centralizing of services as well as flexibility of patient care systems as catalysts for Australian nurses’ decreased job satisfaction and eventual resignation. The structure of services posed a barrier to job satisfaction largely due to differing values between the nurses and organizations regarding patient care and resource distribution. Due to expectations of holistic patient care, American NPs studied in Shea [42] were described to “work the system”, or attend to patients outside of the organizations’ schedules, in an effort to provide continuity of care to patients and maintain job satisfaction. Tham et al. [43] reported a linkage between enhanced service delivery, including a widened range of services and increased inter-professional cooperation, and high levels of job satisfaction for PHC staff in Australia.

#### 4.2.4. Reward and Professional Development

Eleven studies mentioned the impact of wages and benefits on job satisfaction [30,32,33,35,36,38,39,40,41,44,45]. Australian AMHWs surveyed in Cosgrave et al. [30] perceived their wages to be insufficient due to a large difference in salary between AMHWs and other health professionals working within community mental health services, a factor contributing to AMHWs’ decreased job satisfaction. Using a 5-point scale, Chinese doctors surveyed in Fang et al. [32] were least satisfied with their wages (Mean ± SD = 1.99 ± 1.09), and Grujicic et al. [35], using a 5-point scale, similarly reported physicians and nurses in Serbia to be least satisfied with reward including bonuses (Mean ± SD = 2.07 ± 1.30) and wages (Mean ± SD = 1.76 ± 1.16). Multiple studies found moderate satisfaction with wages although health care workers consistently reported the least satisfaction with wages relative to other studied organizational climate factors. Filipova [33], using a 5-point scale, found American PAs to be least satisfied with wages (Mean ± SD = 3.64 ± 0.77), as did Liu et al. [41] for Chinese health care workers (Mean ± SD = 2.65 ± 0.74). Wang et al. [44], using a 5-point scale, determined Chinese PCPs to experience the least job satisfaction with job rewards, including wages and benefits (Mean ± SD = 2.93 ± 0.38). Leutgeb [40], using a 7-point scale, determined GPs in Germany to be least satisfied with income (Mean ± SD = 3.69 ± 1.6), and reported a strong correlation between income and job satisfaction. In contrast to other studies, Nojima et al. [45] found physicians in Japan to experience the highest degree of job satisfaction with their wages on a 4-point scale (Mean ± SD = 2.4 ± 0.7). Gu et al. [36] further found a significant and strong association between job satisfaction and organizational management, which encapsulated personal income, for physicians in China (β = 0.64, *p* < 0.001).

Six studies assessed the influence of promotion opportunities on job satisfaction [30,32,35,36,38,44]. Promotion opportunities largely were viewed as a barrier to job satisfaction for health care workers across studies: in Cosgrave et al. [30], AMHWs in Australia experienced decreased job satisfaction due to limited career opportunities for individuals with exclusively a Bachelor of Health Sciences (Mental Health) degree. Moderate job satisfaction with promotion opportunities was frequently reported, however health care workers widely experienced low satisfaction with promotion opportunities compared to other analyzed organizational components: Fang et al. [32] reported the least satisfaction with chances for advancement among doctors in China on a 5-point scale (Mean ± SD = 2.21 ± 0.91). Using a 5-point scale, Grujicic et al. [35] determined physicians and nurses in Serbia to experience moderate, however comparatively low, satisfaction with promotion and advancement (Mean ± SD = 2.86 ± 1.38), and using a 7-point scale, Izquierdo-Condoy et al. [38] reported physicians in Ecuador to similarly rate their satisfaction with opportunities for promotion low (Mean ± SD = 3.3 ± 1.7). Wang [44], also using a 7-point scale, found PCPs in China to experience moderate job satisfaction with organizational management including promotion opportunities (Mean ± SD = 3.45 ± 0.77). A significant association was additionally established between job satisfaction and job rewards, including job promotion, for physicians in China in Gu et al. [36] (β = 0.29, *p* < 0.001).

Five studies discussed the impact of training and continuing education opportunities as a facilitator of and barrier to job satisfaction [30,33,37,38,45]. The AMHWs interviewed in Cosgrave et al. [30] reported that their limited training and educational qualifications posed a challenge to career development, negatively influencing their job satisfaction. Izquierdo-Condoy et al. [38] reported low satisfaction with training opportunities on a 7-point scale for physicians in Ecuador (Mean ± SD = 3.4 ± 1.7), and Nojima et al. [45], using a 4-point scale, found physicians in Japan to experience the least amount of satisfaction with their opportunity for professional development, including continuing education opportunities (Mean ± SD = 1.2 ± 0.9). Hartung and Miller [37] rather examined continuing education in the context of American nurse managers’ management and communication abilities, and reported continuing education to help develop staffs’ perspectives and overall job satisfaction. Filipova [33] additionally identified a statistically significant correlation between job practice, including continuing education opportunities, and job satisfaction.

**Table 1:** Qualitative studies: key findings on the organizational climate factors of job satisfaction.

First Author (Year)	Title	Country	Health Setting	Methods, Sample Size	Population Description	Key Findings on Organizational Climate and Job Satisfaction/Dissatisfaction (JS & JD)
Bragg (2014) [29]	Degree of value alignment- a grounded theory of rural nurse resignations	Australia	Rural New South Wales hospitals	Semi-structured interviews N = 12	Nurses	(1) Shared values (with nurses and the organization) result in sufficient levels of JS: include perceptions of necessary support and resources required to provide high standard of patient care (i.e., good communication and budget) (2) Nurses’/organizations’ differing values contributes to JD: emphasis on organizational issues (i.e., low expenditure) may prevent nurses’ goal for high standard of patient care; catalysts for JD include restructuring of services, negative management culture, bullying, insufficient staff, etc. (3) Resigning tied to powerlessness, compromised integrity, lack of connectedness
Cosgrave (2017) [30]	Factors affecting job satisfaction of Aboriginal mental health workers working in community mental health in rural and remote New South Wales	Australia	Rural and remote community mental health services in New South Wales	Semi-structured interviews N = 5	Mental health workers	Factors found to influence JS: (1) Difficulties being accepted into the team and organization due to role clarity issues (2) Culturally specific work challenges (i.e., expectation to be Aboriginal “cultural consultant”) (3) Professional differences and inequality relating to differences in qualifications and opportunities for advancement
Hartung (2018) [37]	Rural nurse managers’ perspectives into better communication practices	United States	Community health settings in central and northcentral Pennsylvania	Semi-structured interviews N = 9	Nurse managers	JS affected by interrelated areas: (1) Context: tone and tools of communication (2) Conditions: issues with loneliness/ communication between staff due to isolated nature of rural practice (3) Core: successful communication going beyond a lack of resources (4) Actions: communication and management strategies to promote a healthy environment
de Oliveira (2019) [31]	Satisfaction and limitation of primary health care nurses’ work in rural areas	Brazil	Rural health units of 3 rural communities in Campina Grande	Semi-structured interviews N = 11	Nurses	(1) Teamwork found to facilitate JS through routine, community, respect, leadership, support, and unity (2) Barriers to JS: work overload (expectation to manage bureaucratic issues), lack of professional recognition by managers (poor managerial support, poor interpersonal relations), and lack of resources (i.e., water, technology)
Shea (2015) [42]	Determined persistence: Achieving and sustaining job satisfaction among nurse practitioners	United States	Nurse practitioner work settings in a rural northeastern state	Open-ended interviews N = 15	Nurse practitioners	JS found to stem partly from reconciling the work environment to make it congruent with the worker’s expectations of professional autonomy and holistic patient care; subprocesses include struggling for acceptance from staff and balancing the work environment (i.e., changes in the health care system)
Tham (2014) [43]	Staff perceptions of primary healthcare service change: influences on staff satisfaction	Australia	Primary health service in a small rural community north of Melbourne	Semi-structured interviews N = 10	General practitioners (2), managers (4), allied health staff (2), nurse (1), administrative officer (1)	JS linked to strong inter-professional collaboration, improved working conditions, enhanced service delivery, and role clarity

JS, job satisfaction; JD, job dissatisfaction.

**Table 2:** Quantitative studies: key findings on the organizational climate factors of job satisfaction.

First Author (Year)	Title	Country	Health Setting	Methods, Sample Size	Population Description	Key Findings on Organizational Climate and Job Satisfaction/Dissatisfaction (JS & JD)
Fang (2014) [32]	Factors that influence the turnover intention of Chinese village doctors based on the investigation results of Xiangyang City in Hubei Province	China	1184 village clinics in Xiangyang City	Survey N = 1889	Doctors	Climate factor by highest to lowest JS score on a 0–4 rating scale (Mean ± SD) (1) Most satisfied with the competence of managers in making decisions (2.87 ± 0.82), chance to do something that makes use of abilities, (2.81 ± 0.77), manner that the boss handles workers (2.76 ± 0.86), and being able to keep busy/ fulfilled (2.73 ± 0.82) (2) Most dissatisfied with work conditions (2.34 ± 0.99), chances for advancement (2.21 ± 0.91), and wages and workload (1.99 ± 1.09)
Filipova (2014) [33]	Factors influencing the satisfaction of rural physician assistants	United States	Physician assistant settings in rural communities in a single midwestern state	Survey N = 414	Physician assistants	Climate factor by highest to lowest JS score on a 1–5 rating scale (Mean ± SD) (1) Most satisfied with autonomy/authority (4.48 ± 0.55) and supervising physician (4.37 ± 0.66) (2) Least satisfied with workload and salary (3.64 ± 0.77) (3) Statistically significant correlation found between importance of job practice (i.e., access to resources, reimbursement, autonomy, continuing medical education opportunities) and job satisfaction factors (4) Statistically significant correlation between importance of socialization (i.e., with peers) and satisfaction with workload/salary
Giles (2017) [34]	How do nurse consultant job characteristics impact on job satisfaction? An Australian quantitative study	Australia	Local Health District in New South Wales	Survey N = 106, 45 located rurally	Nurse consultants	Hierarchical regression analysis (1) Role clarity has significant positive impact on JS (β = 0.20, t = 2.19, *p* = 0.03, CI = 0.03, 0.56) (2) Role conflict has significant negative impact on JS (β= −0.23, t = −2.48, *p* = 0.01, CI = −0.4, −0.05) (3) Job autonomy has significant positive impact on JS (β = 0.22, t = 2.51, *p* = 0.01, CI = 0.04, 0.35) (4) Job support has significant positive impact on JS (β = 0.27, t = 3.02, *p* < 0.01, CI = 0.05, 0.24)
Grujicic (2016) [35]	Work motivation and job satisfaction of health workers in urban and rural areas	Serbia	2 Urban health facilities in Belgrade and 2 rural health facilities in Valjevo	Survey N = 832, 436 located rurally	Physicians (91), nurses (345)	Climate factor by highest to lowest rural fulfillment/JS score on a 1–5 Likert scale (Mean ± SD) (1) Highest JS with autonomy (3.61 ± 1.32), professional supervision (3.49 ± 1.43), personal qualities of immediate supervisors (3.48 ± 1.43), and goals of the institution (3.29 ± 1.41) (2) Lowest JS with promotion and advancement (2.86 ± 1.38), reward including time off and bonuses (2.07 ± 1.30), and income (1.76 ± 1.16)
Gu (2019) [36]	Job satisfaction of certified primary care physicians in rural Shandong Province, China: a cross-sectional study	China	Primary health care facilities in rural Shandong province	Survey N = 495	Physicians	Multivariate linear regression analysis of multi-item scales using standardized coefficients Factors significantly associated with JS from highest to lowest relation: (1) organizational management including logistical support and personal income (β = 0.64), (2) external environment including government/supervision of care (β = 0.43), (3) internal environment including cooperation and peer/supervisor relationships (β = 0.36), (4) working conditions including medical equipment and talented staff (β = 0.34), (5) job rewards including job promotion (β = 0.29) (6) competency behaviors including decision-making (β = 0.25), (7) job description including workload (β = 0.11) *p* < 0.001
Izquierdo-Condoy (2023) [38]	Job satisfaction and self-perceptions among Ecuadorian medical doctors during their compulsory rural community social service: a countrywide cross-sectional analysis	Ecuador	Rural physician settings nationwide	Survey N = 247	Physicians	JS score by climate factor on a 1–7 rating scale (Mean ± SD) (1) Highest JS related to participation in organizational and departmental decisions (4.5 ± 1.6) (2) Higher degree of JD related to equality and fairness of treatment (3.4 ± 1.8), training opportunities (3.4 ± 1.7), and opportunities for promotion (3.3 ± 1.7)
Lennon (2019) [39]	Attracting junior doctors to rural centres: a national study of work-life conditions and satisfaction	Australia	Metropolitan and rural hospitals nationwide	Survey N = 4581, 773 located rurally	Junior doctors	Climate factor by highest to lowest JS score (% satisfied) (1) Most satisfied with colleagues and fellow workers (91.1%), amount of responsibility (83.2%), opportunity to use abilities (80.0%), working conditions (77.2%), recognition for good work (67.7%), ability to choose own work method (65.9%) (2) Least satisfied with unpredictable work hours (37.9%) and time off (22.9%)
Leutgeb (2018) [40]	Job satisfaction and stressors for working in out-of-hours care—a pilot study with general practitioners in a rural area of Germany	Germany	General practitioner settings in a rural region of Hesse	Survey N = 131	General practitioners	Climate factor by highest to lowest JS score on 1–7 Likert scale (1) Most satisfied with colleagues and fellow workers (5.28 ± 1.2) and freedom of working method (5.05 ± 1.5) (2) Least satisfied with income (3.69 ± 1.6) and hours of work (3.6 ± 1.6) (3) Strong correlation found between JS and amount of variety in job, opportunity to use abilities, freedom of working method, amount of responsibility, physical working conditions, hours of work, income, and recognition for work, and workload in out-of-hours care (4) Linear regression analysis found association between variety in job (β = 0.23, *p* = 0.05), modification of current OOHC-organization and JS (β= −0.28, *p* = 0.008)
Liu (2019) [41]	Job satisfaction, work stress, and turnover intentions among rural health workers: a cross-sectional study in 11 western provinces of China	China	Medical institutions located in 11 rural western provinces	Survey N = 5046	Doctors, nurses, pharmacists, etc. (some unlisted)	Climate/work stress factor by highest to lowest JS score on 1–5 Likert scale (Mean ± SD) (1) Highest degree of JS with social recognition satisfaction (3.71 ± 0.78) (2) Lowest degree of JS with reward satisfaction including wages (2.65 ± 0.74)
Nojima (2015) [45]	Job and life satisfaction and preference of future practice locations of physicians on remote islands in Japan	Japan	Physician settings on the rural & remote Oki islands	Survey N = 49	Physicians	Climate factor by highest to lowest JS score on a 0–3 Likert scale (Mean ± SD) (1) Highest degree of JS with salary (2.4 ± 0.7) and teamwork (2.4 ± 0.6) (2) Lowest degree of JS with providing medical clerks (1.3 ± 1.0) and opportunity of professional development (1.2 ± 0.9)
Waddimba (2016) [46]	Frequency of satisfaction and dissatisfaction with practice among rural-based, group-employed physicians and non-physician practitioners	United States	Rural integrated health services in 9 counties of central New York	Survey N = 308	Doctors (182), advanced-practice clinician (126)	Multivariable inflated beta regression models of satisfaction/dissatisfaction with practice: (1) Higher gratification of relatedness needs (b = 0.19, t = 2.49, *p* = 0.01, CI = 0.04, 0.34) linked to increased frequency of JS, heavier workload (b = −0.34, t = −5.65, *p* < 0.0001, CI = −0.46, −0.22) associated with decreased likelihood of frequent JS (2) Heavier workload (b = −1.03, t = −5.44, *p* < 0.0001, CI = −1.41, −0.65) associated with decreased likelihood of being highly satisfied (3) Fulfilled relatedness needs (b = −0.24, t = −4.12, *p* < 0.001, CI = −0.36, −0.13) associated with less frequent JD and higher workload linked to more frequent JD (b = 0.27, t = 4.55, *p* < 0.0001, CI = 0.15, 0.39) (4) Fulfilled autonomy needs (b = 0.36, t = 3.76, *p* < 0.001, CI = 0.17, 0.55) associated with increased variation in JD frequency
Wang (2020) [44]	Job satisfaction, burnout, and turnover intention among primary care providers in rural China: results from structural equation modeling	China	Township health centers in 3 counties in Shandong province	Survey N = 1148	Doctors (699), nurses (136), public health providers (313)	Climate factor by highest to lowest JS score on a 1–5 Likert scale (Mean ± SD) (1) Most JS with medical practicing environment satisfaction including peer relationship and support among departments (3.81 ± 0.64) (2) Least JS with organizational management including feedback, inclusion in decisions, reward, and promotion (3.45 ± 0.77) and job rewards satisfaction including wages and benefits (2.93 ± 0.38)

CI, confidence interval; JS, job satisfaction; JD, job dissatisfaction; SD, standard deviation.

## 5. Discussion

As previous literature has highlighted urban areas and individual professions, this scoping review is the first to establish the components of organizational climate which influence job satisfaction in rural health care workplaces in upper-middle-income and high-income countries. Taking into account the frequency of organizational climate components across studies, as well as their reported influence on job satisfaction, our findings suggest that thirteen organizational climate components were influential as facilitators of and/or barriers to rural health workers’ job satisfaction. These components fall within four overarching categories: interpersonal relationships, individual responsibilities, organizational structure and planning, and reward and professional development. Among the identified components, relationships with leadership, peer relationships, autonomy and freedom of working method, availability and quality of resources, workload including the ability to remain busy but without overwhelming demands, and wages and benefits were the most consistently reported across studies, suggesting the importance of these factors across contexts and professions. Due to the diversity of studies relating to study design, methods, and workplace environment, we cannot conclude to what extent each organizational climate component influences job satisfaction, and we refer to individual studies for examples of organizational climate influence.

Herzberg’s Two-Factor Theory posits that two sets of factors influence workplace attitudes: motivators—factors like autonomy and recognition whose presence can lead to job satisfaction—and hygiene factors—external factors like wages or resources which do not facilitate job satisfaction but can contribute to job dissatisfaction [47]. However, the bidirectional nature of the components identified in this review, each capable of functioning as either a facilitator of or barrier to job satisfaction depending on its presence or scale, does not fully align with this framework. Organizational climate components appear to operate along a continuum, suggesting this commonly used theoretical model of job satisfaction may be inadequate in explaining rural health care workers’ experiences.

Many organizational climate components and sub-components identified in this review were previously found in reviews concerning urban health workplaces: leadership styles, workers’ identification with their organizations’ goals, peer relationships, autonomy, role conflict, resource adequacy, hours of work, salary, promotion opportunities, and education opportunities have all been seen to influence job satisfaction in primarily urban nursing settings [1,16,18,48,49]. While this review cannot determine which organizational climate components are most pertinent to rural workplaces compared to urban workplaces, Weinhold et al. [50] formerly linked issues such as geographic isolation, lack of cultural awareness, lack of resources, high workload, and inadequate wages to shortages of rural health care workers. The features of rural settings place health care workers at unique risk by amplifying the salience of organizational climate components: geographic isolation can limit one’s ability to develop strong relationships with leadership and peers, staff and resource scarcity can heighten workload pressures and prevent capable workers from providing care, individual or small team work can prevent development of peer support systems, distance from larger professional networks or institutions can limit professional advancement, and limited cultural awareness can heighten perceptions of inequity [51]. Alternatively, rurality may be protective in some contexts; small team sizes, for example, can provide workers with the opportunity to form the close-knit, supportive team shown to facilitate job satisfaction [43]. Further research surrounding the unique nature of rural work environments is strongly encouraged to understand the impact of rurality on job satisfaction.

This review serves to inform rural health organizations of the organizational climate components which influence job satisfaction, providing a basic framework which organizations may utilize in workplace initiatives. Several reviews have examined the impact of workplace interventions on health care workers’ health and wellbeing, however few apply to rural settings alone and focus largely on individual-level interventions. McKennon et al. [52] identified interpersonal and organizational interventions such as peer consultation groups for support and use of community volunteers as care partners for reduced workload to be effective in rural contexts, while other reviews non-specific to rural contexts have identified redesign of patient flow, investment in equipment, reductions in shift duration, leadership support mechanisms, and other interventions to improve health care worker job satisfaction, burnout, and turnover [53,54]. Shiri et al. [55] further reports that as workplace interventions frequently have short-term positive effects on health and wellbeing, they must be implemented as routine programs. Organizations should draw on existing evidence on effective interventions to address the thirteen organizational climate components, with priority areas for intervention including relationships with leadership, peer relationships, autonomy and freedom of working method, availability and quality of resources, workload and the ability to remain busy, and wages and benefits. It is, however, recognized that rural health workplaces are not homogenous, and initiatives to address job satisfaction require consideration of the needs within individual workplaces.

## 6. Limitations

Several limitations were identified in the process of this review. Our search strategy may not have found all relevant studies, in part due to the search of only four bibliographic databases (PubMed, PsycINFO, CINAHL, and Google Scholar). The screened results of Google Scholar were limited to only the first ten pages, and while preliminary inspection of following pages found no additional relevant sources, some sources meeting criteria may not have been captured by this approach. Relevant sources in a non-English language would also not be captured due to the English language restriction within our criteria. The exclusion of gray literature may have skewed results as studies reporting significant associations between organizational climate factors and job satisfaction are more likely to be published. While title and abstract screening was completed by two or more authors to reduce bias in study selection, full-text review and data extraction were conducted by a single author, which while providing consistency could result in bias in the full-text review and data extraction processes. Furthermore, while not completing a quality assessment reflects the exploratory nature of the review, the lack of formal quality assessment means that the relative strength of individual studies was not determined. Thus, components supported by methodologically weaker studies are included alongside those supported by more robust evidence.

The wide range of study topics and methods created difficulties in directly comparing studies and drawing concrete conclusions about the organizational climate components influencing rural job satisfaction. Seven studies did not focus exclusively on organizational climate and job satisfaction in rural areas: rural job satisfaction was examined in the context of related issues including turnover intention, burnout, work-life conditions, or job motivation and satisfaction in urban areas [29,32,35,39,41,44,45]. There was additional difficulty in comparing findings due to a lack of standardization; multiple different questionnaires were used across studies, excluding the modified Warr-Cook-Wall job satisfaction scales utilized in both Lennon et al. [39] and Leutgeb et al. [40] Further, due to there being no universal definition of “rurality” and a lack of explicit definitions provided by included papers, definitions of “rural” likely vary across studies. Studies explicitly describing workplace environments as located in rural regions were included, however this strategy may not have identified all relevant studies that specified low population size, low population density, or geographic isolation. This review did not conduct gender-specific or professional subgroup analyses given the heterogeneity of included studies, and the extent to which organizational climate factors may be experienced differently across genders or roles is unclear. Doctors and nurses comprised the largest studied professional groups, which may skew results towards their experiences rather than rural health care workers as a whole. Lastly, as the majority of studies were conducted among small samples in Australia, China, and the United States, findings cannot be accurately generalized to the country level or to upper-middle-income and high-income countries more broadly.

## 7. Conclusions

Fostering job satisfaction in health care workplaces is critical to health and performance outcomes for rural health workers, organizations, and communities alike [1,2,3,4,5,6,7]. Relationships with leadership, peer relationships, autonomy and freedom of working method, availability and quality of resources, workload and the ability to remain busy, and wages and benefits emerged as the most consistently identified components impacting job satisfaction across studies. Rural health care organizations are encouraged to prioritize these components and implement participatory, communicative leadership structures, foster team cohesion and support, develop health care worker autonomy and limit inefficient oversight structures, address resource gaps, coordinate with workers to determine manageable workloads, and implement competitive wages and benefits. As many of these factors—particularly peer support, resources, workload, and wages—are structurally constrained in rural settings, broader policy-level responses including rural incentive schemes and targeted funding may be required alongside organizational initiatives. In addition to analyzing a wider range of countries—including low and lower-middle income countries—and performing gender and role-specific analyses to improve understanding of job satisfaction across contexts, further research is required to establish associations between job satisfaction and individual organizational climate components. Moreover, organizational initiatives targeting job satisfaction should be evaluated through longitudinal designs to establish effective methods by which organizations can foster health workers’ job satisfaction in the future.

## Data Availability

No new data were created or analyzed in this study.

## Supplementary Materials

The following supporting information can be downloaded at: https://www.mdpi.com/article/10.3390/healthcare14091238/s1, Supplementary Material S1: Preferred Reporting Items for Systematic reviews and Meta-Analyses extension for Scoping Reviews (PRISMA-ScR) Checklist; Supplementary Material S2: Search Strategy

## Abbreviations

OSH	Occupational Safety and Health
PCC	population/concept/context
JBI	Joanna Briggs Institute
PRISMA-ScR	Preferred Reporting Items for Systematic reviews and Meta-Analyses extension for Scoping review
JS	job satisfaction
JD	job dissatisfaction
PA	physician assistant
NC	nurse consultant
AMHW	Aboriginal mental health worker
PCP	primary care provider
NP	nurse practitioner
GP	general practitioner
PHC	primary health care
