# Supplementary material for: Organizational Climate Factors Influencing Job Satisfaction in Rural Health Care Workplaces in Upper-Middle-Income and High-Income Countries: A Scoping Review
Source: Healthcare (Basel). 2026 May 4;14(9):1238. doi: 10.3390/healthcare14091238 (PMC13164284; doi:10.3390/healthcare14091238)
Supplement: Supplementary file 1 [file healthcare-14-01238-s001.zip › Supplementary file 2- Org climate review [GM.ST] (1).pdf]

Organizational climate factors influencing job satisfaction in rural health care workplaces in upper-middle-income and high-income countries: A scoping review

Healthcare

Ginger Minahan[1,2] and Sandra Thompson[2]

1. Department of Global Health, Georgetown University (corresponding author: gem2174@cumc.columbia.edu)

2. Western Australian Centre for Rural Health, University of Western Australia

Supplementary Material S2: Search Strategy

Database: PubMed

1. "Health Personnel"[Mesh]
2. "Health Workforce"[Mesh]
3. 1 OR 2
4. “Job satisfaction” [Mesh]
5. “psychological wellbeing”
6. “psychological well being”
7. 4 OR 5 OR 6
8. rural[tw]
9. "Rural Health Services"[Mesh]
10. "Rural Population"[Mesh]
11. remote[tw]
12. regional[tw]
13. 8 OR 9 OR 10 OR 11 OR 12
14. organization*
15. organisation*
16. work environment
17. “Organizational Culture” [Mesh]
18. organizational climate
19. organisational climate
20. organizational support
21. organisational support
22. 14 OR 15 OR 16 OR 17 OR 18 OR 19 OR 20 OR 21
23. 3 AND 7 AND 13 AND 22
24. limit 23 to (last 10 years, humans, English)

Database: OVID APA PsycInfo

1. exp *Health Personnel/
2. health workforce.mp
3. 1 OR 2
4. exp *Job satisfaction/
5. “psychological wellbeing”.mp
6. “psychological well being”.mp
7. 4 OR 5 OR 6
8. rural.mp
9. exp *Rural Health/
10. exp *Rural Environments/
11. remote.mp
12. regional.mp
13. 8 OR 9 OR 10 OR 11 OR 12
14. exp *Organizational climate/
15. organization*.mp
16. organisation*.mp
17. work environment.mp
18. workplace culture.mp
19. organizational support.mp
20. organisational support.mp
21. 14 OR 15 OR 16 OR 17 OR 18 OR 19 OR 20
22. 3 AND 7 AND 13 AND 21
23. limit 22 to (last 10 years, humans, English)

Database: CINAHL

1. MM Health Personnel+
2. health workforce
3. 1 OR 2
4. MM Job Satisfaction+
5. “psychological wellbeing”
6. MM “Psychological Well-being”+
7. 4 OR 5 OR 6
8. MM Rural Areas+
9. MM Rural Health Services+
10. MM Rural Population+
11. AB remote
12. AB regional
13. 8 OR 9 OR 10 OR 11 OR 12
14. organization*
15. organisation*
16. work environment
17. MM Organizational Culture+
18. organizational climate
19. organisational climate
20. organizational support
21. organisational support
22. 14 OR 15 OR 16 OR 17 OR 18 OR 19 OR 20 OR 21
23. 3 AND 7 AND 13 AND 22
24. limit 23 to (last 10 years, humans, English)

Database: Google Scholar

1. health personnel
2. health workforce
3. 1 OR 2
4. job satisfaction
5. “psychological wellbeing”
6. “psychological well being”
7. 4 OR 5 OR 6
8. rural
9. “rural health services”
10. “rural population”
11. remote
12. regional
13. 8 OR 9 OR 10 OR 11 OR 12
14. organization
15. organisation
16. work environment
17. workplace culture
18. organizational climate
19. organisational climate
20. organizational support
21. organisational support
22. 14 OR 15 OR 16 OR 17 OR 18 OR 19 OR 20 OR 21
23. 3 AND 7 AND 13 AND 22
24. limit 23 to (last 10 years, first 10 pages of results)
